# Decomposing Probabilistic Lambda-Calculi

**DOI:** 10.1007/978-3-030-45231-5_8

**Published:** 2020-04-17

**Authors:** Ugo Dal Lago, Giulio Guerrieri, Willem Heijltjes

**Affiliations:** 8 ENS/CNRS, Université Paris-Saclay, Cachan, France; 9 University of Duisburg-Essen, Duisburg, Germany; 10 Dipartimento di Informatica - Scienza e Ingegneria, Università di Bologna, Bologna, Italy; 11 Department of Computer Science, University of Bath, Bath, UK

## Abstract

A notion of probabilistic lambda-calculus usually comes with a prescribed reduction strategy, typically call-by-name or call-by-value, as the calculus is non-confluent and these strategies yield different results. This is a break with one of the main advantages of lambda-calculus: confluence, which means results are independent from the choice of strategy. We present a probabilistic lambda-calculus where the probabilistic operator is decomposed into two syntactic constructs: a generator, which represents a probabilistic event; and a consumer, which acts on the term depending on a given event. The resulting calculus, the Probabilistic Event Lambda-Calculus, is confluent, and interprets the call-by-name and call-by-value strategies through different interpretations of the probabilistic operator into our generator and consumer constructs. We present two notions of reduction, one via fine-grained local rewrite steps, and one by generation and consumption of probabilistic events. Simple types for the calculus are essentially standard, and they convey strong normalization. We demonstrate how we can encode call-by-name and call-by-value probabilistic evaluation.

## 1 Introduction

Probabilistic lambda-calculi [24,22,17,11,18,9,15] extend the standard lambda-calculus with a probabilistic choice operator $N \oplus_p M$, which chooses $N$ with probability $p$ and $M$ with probability $1-p$ (throughout this paper, we let $p$ be $1/2$ and will omit it). Duplication of $N \oplus M$, as is wont to happen in lambda-calculus, raises a fundamental question about its semantics: do the duplicate occurrences represent *the same* probabilistic event, or *different* ones with the same probability? For example, take the term $\top \oplus \bot$ that represents a coin flip between boolean values *true* $\top$ and *false* $\bot$. If we duplicate this term, do the copies represent two distinct coin flips with possibly distinct outcomes, or do these represent a single coin flip that determines the outcome for both copies? Put differently again, when we duplicate $\top \oplus \bot$, do we duplicate the *event*, or only its *outcome*?

In probabilistic lambda-calculus, these two interpretations are captured by the evaluation strategies of call-by-name ($\to_{\mathsf{cbn}}$), which duplicates events, and call-by-value ($\rightarrow_{\mathsf{cbv}}$), which evaluates any probabilistic choice before it is duplicated, and thus only duplicates outcomes. Consider the following example, where $=$ tests equality of boolean values.

$$\top \quad {}_{\mathsf{cbv}}\twoheadleftarrow \quad (\lambda x.\, x = x)(\top \oplus \bot) \quad \twoheadrightarrow_{\mathsf{cbn}} \quad \top \oplus \bot$$

This situation is not ideal, for several, related reasons. Firstly, it demonstrates how probabilistic lambda-calculus is non-confluent, negating one of the central properties of the lambda-calculus, and one of the main reasons why it is the prominent model of computation that it is. Secondly, it means that a probabilistic lambda-calculus must derive its semantics from a prescribed reduction strategy, and its terms only have meaning in the context of that strategy. Thirdly, combining different kinds of probabilities becomes highly involved [15], as it would require specialized reduction strategies. These issues present themselves even in a more general setting, namely that of commutative (algebraic) effects, which in general do not commute with copying.

We address these issues by a decomposition of the probabilistic operator into a *generator* $\boxed{a}$ and a *choice* $\overset{a}{\oplus}$, as follows.

$$N \oplus M \quad \overset{\Delta}{=} \quad \boxed{a}.\, N \overset{a}{\oplus} M$$

Semantically, $\boxed{a}$ represents a probabilistic event, that generates a boolean value recorded as $a$. The choice $N \overset{a}{\oplus} M$ is simply a conditional on $a$, choosing $N$ if $a$ is false and $M$ if $a$ is true. Syntactically, $a$ is a boolean variable with an occurrence in $\overset{a}{\oplus}$, and $\boxed{a}$ acts as a probabilistic quantifier, binding all occurrences in its scope. (To capture a non-equal chance, one would attach a probability $p$ to a generator, as $\boxed{a}_p$, though we will not do so in this paper.)

The resulting *probabilistic event lambda-calculus* $\Lambda_{\mathsf{PE}}$, which we present in this paper, is confluent. Our decomposition allows us to separate duplicating an *event*, represented by the generator $\boxed{a}$, from duplicating only its *outcome* $a$, through having multiple choice operators $\overset{a}{\oplus}$. In this way our calculus may interpret both original strategies, call-by-name and call-by-value, by different translations of standard probabilistic terms into $\Lambda_{\mathsf{PE}}$: call-by-name by the above decomposition (see also Section 2), and call-by-value by a different one (see Section 7). For our initial example, we get the following translations and reductions.

$$\mathsf{cbn}: \quad (\lambda x.\, x{=}x)(\boxed{a}.\, \top \overset{a}{\oplus} \bot) \quad \rightarrow_\beta \quad (\boxed{a}.\, \top \overset{a}{\oplus} \bot) = (\boxed{b}.\, \top \overset{b}{\oplus} \bot) \quad \twoheadrightarrow \quad \top \oplus \bot \qquad (1)$$

$$\mathsf{cbv}: \quad \boxed{a}.\, (\lambda x.\, x{=}x)(\top \overset{a}{\oplus} \bot) \quad \rightarrow_\beta \quad \boxed{a}.\, (\top \overset{a}{\oplus} \bot) = (\top \overset{a}{\oplus} \bot) \quad \twoheadrightarrow \quad \top \qquad (2)$$

We present two reduction relations for our probabilistic constructs, both independent of beta-reduction. Our main focus will be on *permutative* reduction (Sections 2, 3), a small-step local rewrite relation which is computationally inefficient but gives a natural and very fine-grained operational semantics. *Projective* reduction (Section 6) is a more standard reduction, following the intuition that $\boxed{a}$ generates a coin flip to evaluate $\overset{a}{\oplus}$, and is coarser but more efficient.

We further prove confluence (Section 4), and we give a system of simple types and prove strong normalization for typed terms by reducibility (Section 5). Omitted proofs can be found in [7], the long version of this paper.

### 1.1 Related Work

Probabilistic $\lambda$-calculi are a topic of study since the pioneering work by Saheb-Djaromi [24], the first to give the syntax and operational semantics of a $\lambda$-calculus with binary probabilistic choice. Giving well-behaved denotational models for probabilistic $\lambda$-calculi has proved to be challenging, as witnessed by the many contributions spanning the last thirty years: from Jones and Plotkin's early study of the probabilistic powerdomain [17], to Jung and Tix's remarkable (and mostly negative) observations [18], to the very recent encouraging results by Goubault-Larrecq [16]. A particularly well-behaved model for probabilistic $\lambda$-calculus can be obtained by taking a probabilistic variation of Girard's coherent spaces [10], this way getting full abstraction [13].

On the operational side, one could mention a study about the various ways the operational semantics of a calculus with binary probabilistic choice can be specified, namely by small-step or big-step semantics, or by inductively or coinductively defined sets of rules [9]. Termination and complexity analysis of higher-order probabilistic programs seen as $\lambda$-terms have been studied by way of type systems in a series of recent results about size [6], intersection [4], and refinement type disciplines [1]. Contextual equivalence on probabilistic $\lambda$-calculi has been studied, and compared with equational theories induced by Böhm Trees [19], applicative bisimilarity [8], or environmental bisimilarity [25].

In all the aforementioned works, probabilistic $\lambda$-calculi have been taken as implicitly endowed with either call-by-name or call-by-value strategies, for the reasons outlined above. There are only a few exceptions, namely some works on Geometry of Interaction [5], Probabilistic Coherent Spaces [14], and Standardization [15], which achieve, in different contexts, a certain degree of independence from the underlying strategy, thus accommodating both call-by-name and call-by-value evaluation. The way this is achieved, however, invariably relies on Linear Logic or related concepts. This is deeply different from what we do here.

Some words of comparison with Faggian and Ronchi Della Rocca's work on confluence and standardization [15] are also in order. The main difference between their approach and the one we pursue here is that the operator ! in their calculus $\Lambda^!_\oplus$ plays *both* the roles of a marker for duplicability and of a checkpoint for any probabilistic choice "flowing out" of the term (*i.e.* being fired). In our calculus, we do not control duplication, but we definitely make use of checkpoints. Saying it another way, Faggian and Ronchi Della Rocca's work is inspired by linear logic, while our approach is inspired by deep inference, even though this is, on purpose, not evident in the design of our calculus.

Probabilistic $\lambda$-calculi can also be seen as vehicles for expressing probabilistic models in the sense of bayesian programming [23,3]. This, however, requires an operator for modeling conditioning, which complicates the metatheory considerably, and that we do not consider here.

Our permutative reduction is a refinement of that for the call-by-name probabilistic $\lambda$-calculus [20], and is an implementation of the equational theory of *(ordered) binary decision trees* via rewriting [27]. Probabilistic decision trees have been proposed with a primitive binary probabilistic operator [22], but not with a decomposition as we explore here.

## 2 The Probabilistic Event $\lambda$-Calculus $\Lambda_{\mathsf{PE}}$

**Definition 1.** The *probabilistic event $\lambda$-calculus* ($\Lambda_{\mathsf{PE}}$) is given by the following grammar, with from left to right: a *variable* (denoted by $x, y, z, \ldots$), an *abstraction*, an *application*, a *(labeled) choice*, and a *(probabilistic) generator*.

$$M, N \quad ::= \quad x \mid \lambda x.N \mid NM \mid N \overset{a}{\oplus} M \mid \boxed{a}.N$$

In a term $\lambda x.M$ the abstraction $\lambda x$ binds the free occurrences of the variable $x$ in its scope $M$, and in $\boxed{a}.N$ the generator $\boxed{a}$ binds the *label* $a$ in $N$. The calculus features a decomposition of the usual probabilistic sum $\oplus$, as follows.

$$N \oplus M \quad \overset{\Delta}{=} \quad \boxed{a}.N \overset{a}{\oplus} M \tag{3}$$

The generator $\boxed{a}$ represents a probabilistic *event*, whose outcome, a binary value $\{0,1\}$ represented by the label $a$, is used by the choice operator $\overset{a}{\oplus}$. That is, $\boxed{a}$ flips a coin setting $a$ to 0 (resp. 1), and depending on this $N \overset{a}{\oplus} M$ reduces to $N$ (resp. $M$). We will use the unlabeled choice $\oplus$ as in (3). This convention also gives the translation from a *call-by-name* probabilistic $\lambda$-calculus into $\Lambda_{\mathsf{PE}}$ (the interpretation of a *call-by-value* probabilistic $\lambda$-calculus is in Section 7).

**Reduction.** Reduction in $\Lambda_{\mathsf{PE}}$ will consist of standard $\beta$-reduction $\rightarrow_\beta$ plus an evaluation mechanism for generators and choice operators, which implements probabilistic choice. We will present two such mechanisms: *projective* reduction $\rightarrow_\pi$ and *permutative* reduction $\rightarrow_{\mathsf{p}}$. While projective reduction implements the given intuition for the generator and choice operator, we relegate it to Section 6 and make permutative reduction our main evaluation mechanism, for the reason that it is more fine-grained, and thus more general.

Permutative reduction is based on the idea that any operator distributes over the labeled choice operator (see the reduction steps in Figure 1), even other choice operators, as below.

$$(N \overset{a}{\oplus} M) \overset{b}{\oplus} P \;\sim\; (N \overset{b}{\oplus} P) \overset{a}{\oplus} (M \overset{b}{\oplus} P)$$

To orient this as a rewrite rule, we need to give priority to one label over another. Fortunately, the relative position of the associated generators $\boxed{a}$ and $\boxed{b}$ provides just that. Then to define $\rightarrow_{\mathsf{p}}$, we will want every choice to belong to some generator, and make the order of generators explicit.

**Definition 2.** The set $\mathsf{fl}(N)$ of *free labels* of a term $N$ is defined inductively by:

$$\mathsf{fl}(x) = \emptyset \qquad \mathsf{fl}(MN) = \mathsf{fl}(M) \cup \mathsf{fl}(N) \qquad \mathsf{fl}(\lambda x.M) = \mathsf{fl}(M)$$
$$\mathsf{fl}(\boxed{a}.M) = \mathsf{fl}(M) \smallsetminus \{a\} \qquad \mathsf{fl}(M \overset{a}{\oplus} N) = \mathsf{fl}(M) \cup \mathsf{fl}(N) \cup \{a\}$$

A term $M$ is *label-closed* if $\mathsf{fl}(M) = \emptyset$.

$$(\lambda x.N)M \rightarrow_\beta N[M/x] \qquad (\beta)$$

$$N \overset{a}{\oplus} N \rightarrow_{\mathsf{p}} N \qquad (\mathsf{i})$$

$$(N \overset{a}{\oplus} M) \overset{a}{\oplus} P \rightarrow_{\mathsf{p}} N \overset{a}{\oplus} P \qquad (\mathsf{c}_1)$$

$$N \overset{a}{\oplus} (M \overset{a}{\oplus} P) \rightarrow_{\mathsf{p}} N \overset{a}{\oplus} P \qquad (\mathsf{c}_2)$$

$$\lambda x.\,(N \overset{a}{\oplus} M) \rightarrow_{\mathsf{p}} (\lambda x.\,N) \overset{a}{\oplus} (\lambda x.\,M) \qquad (\oplus\lambda)$$

$$(N \overset{a}{\oplus} M)P \rightarrow_{\mathsf{p}} (NP) \overset{a}{\oplus} (MP) \qquad (\oplus\mathsf{f})$$

$$N(M \overset{a}{\oplus} P) \rightarrow_{\mathsf{p}} (NM) \overset{a}{\oplus} (NP) \qquad (\oplus\mathsf{a})$$

$$(N \overset{a}{\oplus} M) \overset{b}{\oplus} P \rightarrow_{\mathsf{p}} (N \overset{b}{\oplus} P) \overset{a}{\oplus} (M \overset{b}{\oplus} P) \qquad (\text{if } a < b) \qquad (\oplus\oplus_1)$$

$$N \overset{b}{\oplus} (M \overset{a}{\oplus} P) \rightarrow_{\mathsf{p}} (N \overset{b}{\oplus} M) \overset{a}{\oplus} (N \overset{b}{\oplus} P) \qquad (\text{if } a < b) \qquad (\oplus\oplus_2)$$

$$\boxed{b}.\,(N \overset{a}{\oplus} M) \rightarrow_{\mathsf{p}} (\boxed{b}.\,N) \overset{a}{\oplus} (\boxed{b}.\,M) \qquad (\text{if } a \neq b) \qquad (\oplus\square)$$

$$\boxed{a}.\,N \rightarrow_{\mathsf{p}} N \qquad (\text{if } a \notin \mathsf{fl}(N)) \qquad (\not\square)$$

$$\lambda x.\,\boxed{a}.\,N \rightarrow_{\mathsf{p}} \boxed{a}.\,\lambda x.\,N \qquad (\square\lambda)$$

$$(\boxed{a}.\,N)M \rightarrow_{\mathsf{p}} \boxed{a}.\,(NM) \qquad (\text{if } a \notin \mathsf{fl}(M)) \qquad (\square\mathsf{f})$$

**Fig. 1.** Reduction Rules for $\beta$-reduction and $\mathsf{p}$-reduction.

From here on, we consider only label-closed terms (we implicitly assume this, unless otherwise stated). All terms are identified up to renaming of their bound variables and labels. Given some terms $M$ and $N$ and a variable $x$, $M[N/x]$ is the capture-avoiding (for both variables and labels) substitution of $N$ for the free occurrences of $x$ in $M$. We speak of a *representative* $M$ of a term when $M$ is not considered up to such a renaming. A representative $M$ of a term is *well-labeled* if for every occurrence of $\boxed{a}$ in $M$ there is no $\boxed{a}$ occurring in its scope.

**Definition 3 (Order for labels).** Let $M$ be a well-labeled representative of a term. We define an *order* $<_M$ for the labels occurring in $M$ as follows: $a <_M b$ if and only if $\boxed{b}$ occurs in the scope of $\boxed{a}$.

For a well-labeled and label-closed representative $M$, $<_M$ is a finite tree order.

**Definition 4.** *Reduction* $\rightarrow = \rightarrow_\beta \cup \rightarrow_{\mathsf{p}}$ in $\Lambda_{\mathsf{PE}}$ consists of $\beta$*-reduction* $\rightarrow_\beta$ and *permutative* or $\mathsf{p}$*-reduction* $\rightarrow_{\mathsf{p}}$, both defined as the contextual closure of the rules given in Figure 1. We write $\twoheadrightarrow$ for the reflexive–transitive closure of $\rightarrow$, and $\twoheadrightarrow\!\!\!\!\!\!\mid\,$ for reduction to normal form; similarly for $\rightarrow_\beta$ and $\rightarrow_{\mathsf{p}}$. We write $=_{\mathsf{p}}$ for the symmetric and reflexive–transitive closure of $\rightarrow_{\mathsf{p}}$.

$$\begin{array}{llll} \boxed{a}.\,(\lambda x.\,x{=}x)(\top \overset{a}{\oplus} \bot) & \rightharpoonup_{\mathsf{p}} & \boxed{a}.\,(\lambda x.\,x{=}x)\top \overset{a}{\oplus} (\lambda x.\,x{=}x)\bot & (\oplus\mathsf{a}) \\ & \twoheadrightarrow_{\beta} & \boxed{a}.\,(\top{=}\top) \overset{a}{\oplus} (\bot{=}\bot) & \\ & = & \boxed{a}.\,\top \overset{a}{\oplus} \top \;\rightharpoonup_{\mathsf{p}}\; \boxed{a}.\,\top \;\rightharpoonup_{\mathsf{p}}\; \top & (\mathsf{i},\not\boxed{}) \end{array}$$

**Fig. 2.** Example Reduction of the cbv-translation of the Term on p. 137.

Two example reductions are (1)-(2) on p. 137; a third, complete reduction is in Figure 2. The crucial feature of p-reduction is that a choice $\overset{a}{\oplus}$ *does* permute out of the argument position of an application, but a generator $\boxed{a}$ does *not*, as below. Since the argument of a redex may be duplicated, this is how we characterize the difference between the *outcome* of a probabilistic event, whose duplicates may be identified, and the event itself, whose duplicates may yield different outcomes.

$$N\,(M \overset{a}{\oplus} P) \;\rightharpoonup_{\mathsf{p}}\; (NM) \overset{a}{\oplus} (NP) \qquad\qquad N\,(\boxed{a}.\,M) \;\not\rightharpoonup_{\mathsf{p}}\; \boxed{a}.\,N\,M$$

By inspection of the rewrite rules in Figure 1, we can then characterize the normal forms of $\rightharpoonup_{\mathsf{p}}$ and $\rightharpoonup$ as follows.

**Proposition 5 (Normal forms).** *The normal forms $P_0$ of $\rightharpoonup_{\mathsf{p}}$, respectively $N_0$ of $\rightharpoonup$, are characterized by the following grammars.*

$$\begin{array}{lcl} P_0 & ::= & P_1 \mid P_0 \oplus P_0' \\ P_1 & ::= & x \mid \lambda x.P_1 \mid P_1\,P_0 \end{array} \qquad\qquad \begin{array}{lcl} N_0 & ::= & N_1 \mid N_0 \oplus N_0' \\ N_1 & ::= & N_2 \mid \lambda x.N_1 \\ N_2 & ::= & x \mid N_2\,N_0 \end{array}$$

## 3 Properties of Permutative Reduction

We will prove strong normalization and confluence of $\rightharpoonup_{\mathsf{p}}$. For strong normalization, the obstacle is the interaction between different choice operators, which may duplicate each other, creating super-exponential growth.[3] Fortunately, Dershowitz's *recursive path orders* [12] seem tailor-made for our situation.

Observe that the set $\Lambda_{\mathsf{PE}}$ endowed with $\rightharpoonup_{\mathsf{p}}$ is a first-order term rewriting system over a countably infinite set of variables and the signature $\Sigma$ given by:

- the binary function symbol $\overset{a}{\oplus}$, for any label $a$;
- the unary function symbol $\boxed{a}$, for any label $a$;
- the unary function symbol $\lambda x$, for any variable $x$;
- the binary function symbol @, letting $@(M, N)$ stand for $MN$.

[3] This was inferred only from a simple simulation; we would be interested to know a rigorous complexity result.

**Definition 6.** Let $M$ be a well-labeled representative of a label-closed term, and let $\Sigma_M$ be the set of signature symbols occurring in $M$. We define $\prec_M$ as the (strict) partial order on $\Sigma_M$ generated by the following rules.

$$\begin{array}{ll} \overset{a}{\oplus} \prec_M \overset{b}{\oplus} & \text{if } a <_M b \\ \overset{a}{\oplus} \prec_M \boxed{b} & \text{for any labels } a, b \\ \boxed{b} \prec_M @, \lambda x & \text{for any label } b \end{array}$$

**Lemma 7.** *The reduction $\rightarrow_{\mathsf{p}}$ is strongly normalizing.*

*Proof.* For the first-order term rewriting system $(\Lambda_{\mathsf{PE}}, \rightarrow_{\mathsf{p}})$ we derive a well-founded recursive path ordering $<$ from $\prec_M$ following [12, p. 289]. Let $f$ and $g$ range over function symbols, let $[N_1, \ldots, N_n]$ denote a multiset and extend $<$ to multisets by the standard multiset ordering, and let $N = f(N_1, \ldots, N_n)$ and $M = g(M_1, \ldots, M_m)$; then

$$N < M \iff \begin{cases} [N_1, \ldots, N_n] < [M_1, \ldots, M_m] & \text{if } f = g \\ [N_1, \ldots, N_n] < [M] & \text{if } f \prec_M g \\ [N] \leq [M_1, \ldots, M_m] & \text{if } f \not\preceq_M g \ . \end{cases}$$

While $\prec_M$ is defined only relative to $\Sigma_M$, reduction may only reduce the signature. Inspection of Figure 1 then shows that $M \rightarrow_{\mathsf{p}} N$ implies $N < M$. □

**Confluence of Permutative Reduction.** With strong normalization, confluence of $\rightarrow_{\mathsf{p}}$ requires only local confluence. We reduce the number of cases to consider, by casting the permutations of $\overset{a}{\oplus}$ as instances of a common shape.

**Definition 8.** We define a *context* $C[\,]$ (with exactly one hole $[\,]$) as follows, and let $C[N]$ represent $C[\,]$ with the hole $[\,]$ replaced by $N$.

$$C[\,] ::= [\,] \mid \lambda x.C[\,] \mid C[\,]M \mid NC[\,] \mid C[\,]\overset{a}{\oplus}M \mid N\overset{a}{\oplus}C[\,] \mid \boxed{a}.\,C[\,]$$

Observe that the six reduction rules $\oplus\lambda$ through $\oplus\Box$ in Figure 1 are all of the following form. We refer to these collectively as $\oplus\star$.

$$C[N\overset{a}{\oplus}M] \rightarrow_{\mathsf{p}} C[N]\overset{a}{\oplus}C[M] \qquad (\oplus\star)$$

**Lemma 9 (Confluence of $\rightarrow_{\mathsf{p}}$).** *Reduction $\rightarrow_{\mathsf{p}}$ is confluent.*

*Proof.* By Newman's lemma and strong normalization of $\rightarrow_{\mathsf{p}}$ (Lemma 7), confluence follows from local confluence. The proof of local confluence consists of joining all critical pairs given by $\rightarrow_{\mathsf{p}}$. Details are in the Appendix of [7]. □

**Definition 10.** We denote the unique $\mathsf{p}$-normal form of a term $N$ by $N_{\mathsf{p}}$.

## 4 Confluence

We aim to prove that $\to\,=\,\to_\beta \cup \to_{\mathsf{p}}$ is confluent. We will use the standard technique of *parallel* $\beta$-reduction [26], a simultaneous reduction step on a number of $\beta$-redexes, which we define via a labeling of the redexes to be reduced. The central point is to find a notion of reduction that is *diamond*, *i.e.* every critical pair can be closed in one (or zero) steps. This will be our *complete* reduction, which consists of parallel $\beta$-reduction followed by $\mathsf{p}$-reduction to normal form.

**Definition 11.** A *labeled* term $P^\bullet$ is a term $P$ with chosen $\beta$-redexes annotated as $(\lambda x. N)^\bullet M$. The unique *labeled $\beta$-step* $P^\bullet \Rightarrow_\beta P_\bullet$ from $P^\bullet$ to the *labeled reduct* $P_\bullet$ reduces every labeled redex, and is defined inductively as follows.

$$(\lambda x. N^\bullet)^\bullet M^\bullet \Rightarrow_\beta N_\bullet[M_\bullet/x] \qquad\qquad N^\bullet M^\bullet \Rightarrow_\beta N_\bullet M_\bullet$$

$$x \Rightarrow_\beta x \qquad\qquad N^\bullet \overset{a}{\oplus} M^\bullet \Rightarrow_\beta N_\bullet \overset{a}{\oplus} M_\bullet$$

$$\lambda x. N^\bullet \Rightarrow_\beta \lambda x. N_\bullet \qquad\qquad \boxed{a}. N^\bullet \Rightarrow_\beta \boxed{a}. N_\bullet$$

A *parallel $\beta$-step* $P \Rightarrow_\beta P_\bullet$ is a labeled step $P^\bullet \Rightarrow_\beta P_\bullet$ for some labeling $P^\bullet$.

Note that $P_\bullet$ is an unlabeled term, since all labels are removed in the reduction. For the empty labeling, $P^\bullet = P_\bullet = P$, so parallel reduction is reflexive: $P \Rightarrow_\beta P$.

**Lemma 12.** *A parallel $\beta$-step $P \Rightarrow_\beta P_\bullet$ is a $\beta$-reduction $P \twoheadrightarrow_\beta P_\bullet$.*

*Proof.* By induction on the labeled term $P^\bullet$ generating $P \Rightarrow_\beta P_\bullet$. □

**Lemma 13.** *Parallel $\beta$-reduction is diamond.*

*Proof.* Let $P^\bullet \Rightarrow_\beta P_\bullet$ and $P^\circ \Rightarrow_\beta P_\circ$ be two labeled reduction steps on a term $P$. We annotate each step with the label of the other, preserved by reduction, to give the span from the doubly labeled term $P^{\bullet\circ} = P^{\circ\bullet}$ below left. Reducing the remaining labels will close the diagram, as below right.

$$P^\circ_\bullet \;\;{}_\beta\!\Leftarrow\;\; P^{\bullet\circ} = P^{\circ\bullet} \;\;\Rightarrow_\beta\;\; P^\bullet_\circ \qquad\qquad P^\circ_\bullet \;\;\Rightarrow_\beta\;\; P_{\bullet\circ} = P_{\circ\bullet} \;\;{}_\beta\!\Leftarrow\;\; P^\bullet_\circ$$

This is proved by induction on $P^{\bullet\circ}$, where only two cases are not immediate: those where a redex carries one but not the other label. One case follows by the below diagram; the other case is symmetric. Below, for the step top right, induction on $N^\bullet$ shows that $N^\bullet[M^\bullet/x] \Rightarrow_\beta N_\bullet[M_\bullet/x]$.

$$\begin{array}{ccccc} (\lambda x. N^{\circ\bullet})^\circ M^{\circ\bullet} & \Rightarrow_\beta & N^\bullet_\circ[M^\bullet_\circ/x] & \Rightarrow_\beta & N_{\circ\bullet}[M_{\circ\bullet}/x] \\ = & & & & = \\ (\lambda x. N^{\bullet\circ})^\circ M^{\bullet\circ} & \Rightarrow_\beta & (\lambda x. N^\circ_\bullet)^\circ M^\circ_\bullet & \Rightarrow_\beta & N_{\bullet\circ}[M_{\bullet\circ}/x] \end{array}$$

□

### 4.1 Parallel Reduction and Permutative Reduction

For the commutation of (parallel) $\beta$-reduction with $\mathsf{p}$-reduction, we run into the minor issue that a permuting generator or choice operator may block a redex: in both cases below, before $\rightarrowtriangle_{\mathsf{p}}$ the term has a redex, but after $\rightarrowtriangle_{\mathsf{p}}$ it is blocked.

$$(\lambda x.\, N \overset{a}{\oplus} M)\, P \rightarrowtriangle_{\mathsf{p}} ((\lambda x.\, N) \overset{a}{\oplus} (\lambda x.\, M))\, P \qquad (\lambda x.\, \boxed{a}.\, N)\, M \rightarrowtriangle_{\mathsf{p}} (\boxed{a}.\, \lambda x.\, N)\, M$$

We address this by an adaptation $\mathrel{\rightarrow\!\!\!\blacktriangleright}_{\mathsf{p}}$ of $\mathsf{p}$-reduction on labeled terms, which is a strategy in $\twoheadrightarrow_{\mathsf{p}}$ that permutes past a labeled redex in one step.

**Definition 14.** A *labeled* $\mathsf{p}$-reduction $N^\bullet \mathrel{\rightarrow\!\!\!\blacktriangleright}_{\mathsf{p}} M^\bullet$ on labeled terms is a $\mathsf{p}$-reduction of one of the forms

$$(\lambda x.\, N^\bullet \overset{a}{\oplus} M^\bullet)^\bullet P^\bullet \twoheadrightarrow_{\mathsf{p}} (\lambda x.\, N^\bullet)^\bullet P^\bullet \overset{a}{\oplus} (\lambda x.\, M^\bullet)^\bullet P^\bullet$$

$$(\lambda x.\, \boxed{a}.\, N^\bullet)^\bullet M^\bullet \twoheadrightarrow_{\mathsf{p}} \boxed{a}.\, (\lambda x.\, N^\bullet)^\bullet M^\bullet$$

or a single $\mathsf{p}$-step $\rightarrowtriangle_{\mathsf{p}}$ on unlabeled constructors in $N^\bullet$.

**Lemma 15.** *Reduction to normal form in* $\mathrel{\rightarrow\!\!\!\blacktriangleright}_{\mathsf{p}}$ *is equal to* $\twoheadrightarrow\!|_{\mathsf{p}}$ *(on labeled terms).*

*Proof.* Observe that $\rightarrowtriangle_{\mathsf{p}}$ and $\mathrel{\rightarrow\!\!\!\blacktriangleright}_{\mathsf{p}}$ have the same normal forms. Then in one direction, since $\mathrel{\rightarrow\!\!\!\blacktriangleright}_{\mathsf{p}} \subseteq \twoheadrightarrow_{\mathsf{p}}$ we have $\mathrel{\twoheadrightarrow\!\!\!\blacktriangleright}\!|_{\mathsf{p}} \subseteq \twoheadrightarrow\!|_{\mathsf{p}}$. Conversely, let $N \twoheadrightarrow\!|_{\mathsf{p}} M$. On this reduction, let $P \rightarrowtriangle_{\mathsf{p}} Q$ be the first step such that $P \mathrel{\not\rightarrow\!\!\!\blacktriangleright}_{\mathsf{p}} Q$. Then there is an $R$ such that $P \mathrel{\rightarrow\!\!\!\blacktriangleright}_{\mathsf{p}} R$ and $Q \rightarrowtriangle_{\mathsf{p}} R$. Note that we have $N \mathrel{\twoheadrightarrow\!\!\!\blacktriangleright}_{\mathsf{p}} R$. By confluence, $R \twoheadrightarrow\!|_{\mathsf{p}} M$, and by induction on the sum length of paths in $\rightarrowtriangle_{\mathsf{p}}$ from $R$ (smaller than from $N$) we have $R \mathrel{\twoheadrightarrow\!\!\!\blacktriangleright}\!|_{\mathsf{p}} M$, and hence $N \mathrel{\twoheadrightarrow\!\!\!\blacktriangleright}\!|_{\mathsf{p}} M$. □

The following lemmata then give the required commutation properties of the relations $\mathrel{\rightarrow\!\!\!\blacktriangleright}_{\mathsf{p}}$, $\twoheadrightarrow\!|_{\mathsf{p}}$, and $\Rrightarrow_{\beta}$. Figure 3 illustrates these by commuting diagrams.

**Lemma 16.** *If* $N^\bullet \mathrel{\rightarrow\!\!\!\blacktriangleright}_{\mathsf{p}} M^\bullet$ *then* $N_\bullet =_{\mathsf{p}} M_\bullet$.

*Proof.* By induction on the rewrite step $\mathrel{\rightarrow\!\!\!\blacktriangleright}_{\mathsf{p}}$. The two interesting cases are:

$$\begin{array}{ccc}
(\lambda x.\, M^\bullet)^\bullet (N^\bullet \overset{a}{\oplus} P^\bullet) & \xrightarrow{\ \mathsf{p}\ } & ((\lambda x.\, M^\bullet)^\bullet N^\bullet) \overset{a}{\oplus} ((\lambda x.\, M^\bullet)^\bullet P^\bullet) \\
\beta \Downarrow & & \Downarrow \beta \\
M_\bullet[(N_\bullet \overset{a}{\oplus} P_\bullet)/x] & \underset{\mathsf{p}}{\dashrightarrow\!\!\!\rightarrow} & M_\bullet[N_\bullet/x] \overset{a}{\oplus} M_\bullet[P_\bullet/x]
\end{array} \qquad (x \in \mathsf{fv}(M))$$

$$\begin{array}{ccc}
(\lambda x.\, M^\bullet)^\bullet (N^\bullet \overset{a}{\oplus} P^\bullet) & \xrightarrow{\ \mathsf{p}\ } & ((\lambda x.\, M^\bullet)^\bullet N^\bullet) \overset{a}{\oplus} ((\lambda x.\, M^\bullet)^\bullet P^\bullet) \\
\beta \Downarrow & & \Downarrow \beta \\
M_\bullet & \underset{\mathsf{p}}{\dashleftarrow} & M_\bullet \overset{a}{\oplus} M_\bullet
\end{array} \qquad (x \notin \mathsf{fv}(M))$$

□

How the critical pairs in the above diagrams are joined shows that we cannot use the Hindley-Rosen Lemma [2, Prop. 3.3.5] to prove confluence of $\rightarrow_\beta \cup \rightarrow_{\mathsf{p}}$.

**Lemma 17.** $N_\bullet =_{\mathsf{p}} N_{\mathsf{p}\bullet}$.

*Proof.* Using Lemma 15 we decompose $N^\bullet \twoheadrightarrow\!\!|_{\mathsf{p}} N^\bullet_{\mathsf{p}}$ as

$$N^\bullet = N^\bullet_1 \rightarrow_{\mathsf{p}} N^\bullet_2 \rightarrow_{\mathsf{p}} \cdots \rightarrow_{\mathsf{p}} N^\bullet_n = N^\bullet_{\mathsf{p}}$$

where $(N_i)_\bullet =_{\mathsf{p}} (N_{i+1})_\bullet$ by Lemma 16. □

## 4.2 Complete Reduction

To obtain a reduction strategy with the diamond property for $\rightarrow$, we combine parallel reduction $\Rightarrow_\beta$ with permutative reduction to normal form $\twoheadrightarrow\!\!|_{\mathsf{p}}$ into a notion of *complete reduction* $\Rightarrow$. We will show that it is diamond (Lemma 19), and that any step in $\rightarrow$ maps onto a complete step of $\mathsf{p}$-normal forms (Lemma 20). Confluence of $\rightarrow$ (Theorem 21) then follows: any two paths $\twoheadrightarrow$ map onto complete paths $\Rightarrow\!\!\!\!\!\!\twoheadrightarrow$ on $\mathsf{p}$-normal forms, which then converge by the diamond property.

**Definition 18.** A *complete* reduction step $N \Rightarrow N_{\bullet\mathsf{p}}$ is a parallel $\beta$-step followed by $\mathsf{p}$-reduction to normal form:

$$N \Rightarrow N_{\bullet\mathsf{p}} \quad := \quad N \Rightarrow_\beta N_\bullet \twoheadrightarrow\!\!|_{\mathsf{p}} N_{\bullet\mathsf{p}} \ .$$

**Lemma 19 (Complete reduction is diamond).** *If $P \Leftarrow N \Rightarrow M$ then for some $Q$, $P \Rightarrow Q \Leftarrow M$.*

*Proof.* By the following diagram, where $M = N_{\circ\mathsf{p}}$ and $P = N_{\bullet\mathsf{p}}$, and $Q = N_{\circ\bullet\mathsf{p}}$. The square top left is by Lemma 13, top right and bottom left are by Lemma 17, and bottom right is by confluence and strong normalization of $\mathsf{p}$-reduction.

$$\begin{array}{ccccc}
N^{\circ\bullet} & \overset{\beta}{\Longrightarrow} & N^\bullet_\circ & \overset{\mathsf{p}}{\twoheadrightarrow\!\!|} & N^\bullet_{\circ\mathsf{p}} \\
{\scriptstyle\beta}\Downarrow & & {\scriptstyle\beta}\Downarrow & & {\scriptstyle\beta}\Downarrow \\
N^\circ_\bullet & \overset{\beta}{\Longrightarrow} & N_{\circ\bullet} & =_{\mathsf{p}} & N_{\circ\mathsf{p}\bullet} \\
{\scriptstyle\mathsf{p}}\downarrow & & =_{\mathsf{p}} & & {\scriptstyle\mathsf{p}}\downarrow \\
N^\circ_{\bullet\mathsf{p}} & \overset{\beta}{\Longrightarrow} & N_{\bullet\mathsf{p}\circ} & \overset{\mathsf{p}}{\twoheadrightarrow\!\!|} & N_{\circ\bullet\mathsf{p}}
\end{array}$$

□

**Lemma 20 ($\mathsf{p}$-Normalization maps reduction to complete reduction).** *If $N \rightarrow M$ then $N_{\mathsf{p}} \Rightarrow M_{\mathsf{p}}$.*

*Proof.* For a $\mathsf{p}$-step $N \rightarrow_{\mathsf{p}} M$ we have $N_{\mathsf{p}} = M_{\mathsf{p}}$ while $\Rightarrow_\beta$ is reflexive. For a $\beta$-step $N \rightarrow_\beta M$ we label the reduced redex in $N$ to get $N^\bullet \Rightarrow_\beta N_\bullet = M$. Then Lemma 17 gives $N_{\mathsf{p}\bullet} =_{\mathsf{p}} M$, and hence $N_{\mathsf{p}} \Rightarrow_\beta N_{\mathsf{p}\bullet} \twoheadrightarrow\!\!|_{\mathsf{p}} M_{\mathsf{p}}$. □

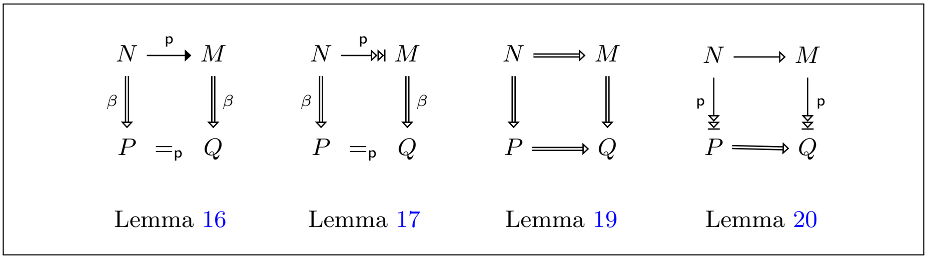

**Fig. 3.** Diagrams for the Lemmata Leading up to Confluence

**Theorem 21.** *Reduction $\to$ is confluent.*

*Proof.* By the following diagram. For the top and left areas, by Lemma 20 any reduction path $N \twoheadrightarrow M$ maps onto one $N_{\mathsf{p}} \Rrightarrow M_{\mathsf{p}}$. The main square follows by the diamond property of complete reduction, Lemma 19.

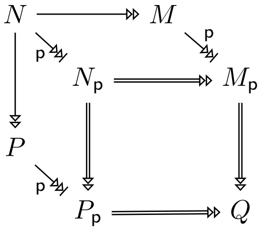

□

## 5 Strong Normalization for Simply-Typed Terms

In this section, we prove that the relation $\to$ enjoys strong normalization in *simply typed* terms. Our proof of strong normalization is based on the classic reducibility technique, and inherently has to deal with label-open terms. It thus make great sense to turn the order $<_M$ from Definition 3 into something more formal, at the same time allowing terms to be label-*open*. This is in Figure 4. It is easy to realize that, of course modulo label $\alpha$-equivalence, for every term $M$ there is at least one $\theta$ such that $\theta \vdash_L M$. An easy fact to check is that if $\theta \vdash_L M$ and $M \to^{\theta} N$, then $\theta \vdash_L N$. It thus makes sense to parametrize $\to$ on a sequence of labels $\theta$, *i.e.*, one can define a family of reduction relations $\to^{\theta}$ on pairs in the form $(M, \theta)$. The set of strongly normalizable terms, and the number of steps to normal forms become themselves parametric:

- The set $SN^{\theta}$ of those terms $M$ such that $\theta \vdash_L M$ and $(M, \theta)$ is strongly normalizing modulo $\to^{\theta}$;
- The function $sn^{\theta}$ assigning to any term in $SN^{\theta}$ the maximal number of $\to^{\theta}$ steps to normal form.

Label Sequences: $\theta \quad ::= \quad \varepsilon \mid a \cdot \theta$

Label Judgments: $\xi \quad ::= \quad \theta \vdash_L M$

Label Rules:

$$\frac{}{\theta \vdash_L x} \qquad \frac{\theta \vdash_L M}{\theta \vdash_L \lambda x.M} \qquad \frac{a \cdot \theta \vdash_L M}{\theta \vdash_L \boxed{a}.\, M}$$

$$\frac{\theta \vdash_L M \quad \theta \vdash_L N}{\theta \vdash_L MN} \qquad \frac{\theta \vdash_L M \quad \theta \vdash_L N \quad a \in \theta}{\theta \vdash_L M \overset{a}{\oplus} N}$$

**Fig. 4.** Labeling Terms

Types: $\tau \quad ::= \quad \alpha \mid \tau \Rightarrow \rho$

Environments: $\Gamma \quad ::= \quad x_1 : \tau_1, \ldots, x_n : \tau_n$

Judgments: $\pi \quad ::= \quad \Gamma \vdash M : \tau$

Typing Rules:

$$\frac{}{\Gamma, x : \tau \vdash x : \tau} \qquad \frac{\Gamma, x : \tau \vdash M : \rho}{\Gamma \vdash \lambda x.M : \tau \Rightarrow \rho} \qquad \frac{\Gamma \vdash M : \tau}{\Gamma \vdash \boxed{a}.\, M : \tau}$$

$$\frac{\Gamma \vdash M : \tau \Rightarrow \rho \quad \Gamma \vdash N : \tau}{\Gamma \vdash MN : \rho} \qquad \frac{\Gamma \vdash M : \tau \quad \Gamma \vdash N : \tau}{\Gamma \vdash M \overset{a}{\oplus} N : \tau}$$

**Fig. 5.** Types, Environments, Judgments, and Rules

$$\frac{L_1 \in SN^\theta \quad \cdots \quad L_m \in SN^\theta}{xL_1 \ldots L_m \in SN^\theta} \qquad \frac{ML_1 \ldots L_m \in SN^\theta \quad NL_1 \ldots L_m \in SN^\theta \quad a \in \theta}{M \overset{a}{\oplus} NL_1 \ldots L_m \in SN^\theta}$$

$$\frac{M[L_0/x]L_1 \ldots L_m \in SN^\theta \quad L_0 \in SN^\theta}{(\lambda x.\, M)L_0 \ldots L_m \in SN^\theta} \qquad \frac{ML_1 \ldots L_m \in SN^{a \cdot \theta} \quad \forall i. a \notin L_i}{(\boxed{a}.\, M)L_1 \ldots L_m \in SN^\theta}$$

**Fig. 6.** Closure Rules for Sets $SN^\theta$

We can now define types, environments, judgments, and typing rules in Figure 5.

Please notice that the type structure is precisely the one of the usual, vanilla, simply-typed $\lambda$-calculus (although terms are of course different), and we can thus reuse most of the usual proof of strong normalization, for example in the version given by Ralph Loader's notes [21], page 17.

**Lemma 22.** *The closure rules in Figure 6 are all sound.*

Since the structure of the type system is the one of plain, simple types, the definition of reducibility sets is the classic one:

$$\begin{aligned} Red_\alpha &= \{(\Gamma, \theta, M) \mid M \in SN^\theta \wedge \Gamma \vdash M : \alpha\}; \\ Red_{\tau \Rightarrow \rho} &= \{(\Gamma, \theta, M) \mid (\Gamma \vdash M : \tau \Rightarrow \rho) \wedge (\theta \vdash_L M) \wedge \\ &\qquad \forall (\Gamma\Delta, \theta, N) \in Red_\tau . (\Gamma\Delta, \theta, MN) \in Red_\rho\}. \end{aligned}$$

Before proving that all terms are reducible, we need some auxiliary results.

**Lemma 23.** *1. If $(\Gamma, \theta, M) \in Red_\tau$, then $M \in SN^\theta$.*
*2. If $\Gamma \vdash xL_1 \dots L_m : \tau$ and $L_1, \dots, L_m \in SN^\theta$, then $(\Gamma, \theta, xL_1 \dots L_m) \in Red_\tau$.*
*3. If $(\Gamma, \theta, M[L_0/x]L_1 \dots L_m) \in Red_\tau$ with $\Gamma \vdash L_0 : \rho$ and $L_0 \in SN^\theta$, then $(\Gamma, \theta, (\lambda x. M)L_0 \dots L_m) \in Red_\tau$.*
*4. If $(\Gamma, \theta, ML_1 \dots L_m) \in Red_\tau$ with $(\Gamma, \theta, NL_1 \dots L_m) \in Red_\tau$ and $a \in \theta$, then $(\Gamma, \theta, (M \overset{a}{\oplus} N)L_1 \dots L_m) \in Red_\tau$.*
*5. If $(\Gamma, a \cdot \theta, ML_1 \dots L_m) \in Red_\tau$ and $a \notin L_i$ for all $i$, then $(\Gamma, \theta, (\boxed{a}. M)L_1 \dots L_m) \in Red_\tau$.*

*Proof.* The proof is an induction on $\tau$: If $\tau$ is an atom $\alpha$, then Point 1 follows by definition, while points 2 to 5 come from Lemma 22. If $\tau$ is $\rho \Rightarrow \mu$, Points 2 to 5 come directly from the induction hypothesis, while Point 1 can be proved by observing that $M$ is in $SN^\theta$ if $Mx$ is itself $SN^\theta$, where $x$ is a fresh variable. By induction hypothesis (on Point 2), we can say that $(\Gamma(x : \rho), \theta, x) \in Red_\rho$, and conclude that $(\Gamma(x : \rho), \theta, Mx) \in Red_\mu$. □

The following is the so-called Main Lemma:

**Proposition 24.** *Suppose $y_1 : \tau_1, \dots, y_n : \tau_n \vdash M : \rho$ and $\theta \vdash_L M$, with $(\Gamma, \theta, N_j) \in Red_{\tau_j}$ for all $1 \leq j \leq n$. Then $(\Gamma, \theta, M[N_1/y_1, \dots, N_n/y_n]) \in Red_\rho$.*

*Proof.* This is an induction on the structure of the term $M$:

- If $M$ is a variable, necessarily one among $y_1, \dots, y_n$, then the result is trivial.
- If $M$ is an application $LP$, then there exists a type $\xi$ such that $y_1 : \tau_1, \dots, y_n : \tau_n \vdash L : \xi \Rightarrow \rho$ and $y_1 : \tau_1, \dots, y_n : \tau_n \vdash P : \xi$. Moreover, $\theta \vdash_L L$ and $\theta \vdash_L P$ we can then safely apply the induction hypothesis and conclude that

$$(\Gamma, \theta, L[\overline{N}/\overline{y}]) \in Red_{\xi \Rightarrow \rho} \qquad (\Gamma, \theta, P[\overline{N}/\overline{y}]) \in Red_\xi \ .$$

By definition, we get

$$(\Gamma, \theta, (LP)[\overline{N}/\overline{y}]) \in Red_\rho \ .$$

- If $M$ is an abstraction $\lambda x. L$, then $\rho$ is an arrow type $\xi \Rightarrow \mu$ and $y_1 : \tau_1, \dots, y_n : \tau_n, x : \xi \vdash L : \mu$. Now, consider any $(\Gamma\Delta, \theta, P) \in Red_\xi$. Our objective is to prove with this hypothesis that $(\Gamma\Delta, \theta, (\lambda x.L[\overline{N}/\overline{y}])P) \in Red_\mu$. By induction hypothesis, since $(\Gamma\Delta, N_i) \in Red_{\tau_i}$, we get that $(\Gamma\Delta, \theta, L[\overline{N}/\overline{y}, P/x]) \in Red_\mu$. The thesis follows from Lemma 23.

- If $M$ is a sum $L \overset{a}{\oplus} P$, we can make use of Lemma 23 and the induction hypothesis, and conclude.
- If $M$ is a generator $\boxed{a}.\,P$, we can make use of Lemma 23 and the induction hypothesis. We should however observe that $a \cdot \theta \vdash_L P$, since $\theta \vdash_L M$. □

We now have all the ingredients for our proof of strong normalization:

**Theorem 25.** *If $\Gamma \vdash M : \tau$ and $\theta \vdash_L M$, then $M \in SN^\theta$.*

*Proof.* Suppose that $x_1 : \rho_1, \ldots, x_n : \rho_n \vdash M : \tau$. Since $x_1 : \rho_1, \ldots, x_n : \rho_n \vdash x_i : \rho_i$ for all $i$, and clearly $\theta \vdash_L x_i$ for every $i$, we can apply Lemma 24 and obtain that $(\Gamma, \theta, M[\overline{x}/\overline{x}]) \in Red_\tau$ from which, via Lemma 23, one gets the thesis. □

## 6 Projective Reduction

Permutative reduction $\rightarrow_{\mathsf{p}}$ evaluates probabilistic sums purely by rewriting. Here we look at a more standard *projective* notion of reduction, which conforms more closely to the intuition that $\boxed{a}$ generates a probabilistic event to determine the choice $\overset{a}{\oplus}$. Using $+$ for an external probabilistic sum, we expect to reduce $\boxed{a}.\,N$ to $N_0 + N_1$ where each $N_i$ is obtained from $N$ by projecting every subterm $M_0 \overset{a}{\oplus} M_1$ to $M_i$. The question is, in what context should we admit this reduction? We first limit ourselves to reducing in *head* position.

**Definition 26.** The *a-projections* $\pi_0^a(N)$ and $\pi_1^a(N)$ are defined as follows:

$$\begin{aligned}
\pi_0^a(N \overset{a}{\oplus} M) &= \pi_0^a(N) & \pi_i^a(\lambda x.\,N) &= \lambda x.\pi_i^a(N) & \\
\pi_1^a(N \overset{a}{\oplus} M) &= \pi_1^a(M) & \pi_i^a(NM) &= \pi_i^a(N)\,\pi_i^a(M) & \\
\pi_i^a(\boxed{a}.\,N) &= \boxed{a}.\,N & \pi_i^a(N \overset{b}{\oplus} M) &= \pi_i^a(N) \overset{b}{\oplus} \pi_i^a(M) & \text{if } a \neq b \\
\pi_i^a(x) &= x & \pi_i^a(\boxed{b}.\,N) &= \boxed{b}.\,\pi_i^a(N) & \text{if } a \neq b.
\end{aligned}$$

**Definition 27.** A *head context* $H[\,]$ is given by the following grammar.

$$H[\,] ::= [\,] \mid \lambda x.\,H[\,] \mid H[\,]N$$

**Definition 28.** *Projective head reduction* $\rightarrow_{\pi\mathsf{h}}$ is given by

$$H[\boxed{a}.\,N] \rightarrow_{\pi\mathsf{h}} H[\pi_0^a(N)] + H[\pi_1^a(N)]\ .$$

We can simulate $\rightarrow_{\pi\mathsf{h}}$ by permutative reduction if we interpret the external sum $+$ by an outermost $\oplus$ (taking special care if the label does not occur).

**Proposition 29.** *Permutative reduction simulates projective head reduction:*

$$H[\boxed{a}.\,N] \twoheadrightarrow_{\mathsf{p}} \begin{cases} H[N] & \text{if } a \notin \mathsf{fl}(N) \\ H[\pi_0^a(N)] \oplus H[\pi_1^a(N)] & \text{otherwise.} \end{cases}$$

*Proof.* The case $a \notin \mathsf{fl}(N)$ is immediate by a $\not\emptyset$ step. For the other case, observe that $H[\boxed{a}.\,N] \twoheadrightarrow_{\mathsf{p}} \boxed{a}.\,H[N]$ by $\Box\lambda$ and $\Box\mathsf{f}$ steps, and since $a$ does not occur in $H[\,]$, that $H[\pi_i^a(N)] = \pi_i^a(H[N])$. By induction on $N$, if $a$ is minimal in $N$ (*i.e.* $a \in \mathsf{fl}(N)$ and $a \le b$ for all $b \in \mathsf{fl}(N)$) then $N \twoheadrightarrow_{\mathsf{p}} \pi_0^a(N) \overset{a}{\oplus} \pi_1^a(N)$. As required,

$$H[\boxed{a}.\,N] \;\twoheadrightarrow_{\mathsf{p}}\; \boxed{a}.\,H[\pi_0^a(N)] \overset{a}{\oplus} H[\pi_1^a(N)] \quad \text{if } a \in \mathsf{fl}(N)\ . \qquad \Box$$

A gap remains between which generators will not be duplicated, which we *should* be able to reduce, and which generators projective head reduction *does* reduce. In particular, to interpret call-by-value probabilistic reduction in Section 7, we would like to reduce under other generators. However, permutative reduction does not permit exchanging generators, and so only simulates reducing in head position. While (independent) probabilistic events are generally considered interchangeable, it is a question whether the below equivalence is desirable.

$$\boxed{a}.\,\boxed{b}.\,N \;\overset{?}{\sim}\; \boxed{b}.\,\boxed{a}.\,N \tag{4}$$

We elide the issue by externalizing probabilistic events, and reducing with reference to a predetermined binary stream $s \in \{0,1\}^{\mathbb{N}}$ representing their outcomes. In this way, we will preserve the intuitions of both permutative and projective reduction: we obtain a qualified version of the equivalence (4) (see (5) below), and will be able to reduce any generator on the *spine* of a term: under (other) generators and choices as well as under abstractions and in function position.

**Definition 30.** The set of *streams* is $\mathbb{S} = \{0,1\}^{\mathbb{N}}$, ranged over by $r, s, t$, and $i \cdot s$ denotes a stream with $i \in \{0,1\}$ as first element and $s$ as the remainder.

**Definition 31.** The *stream labeling* $N^s$ of a term $N$ with a stream $s \in \mathbb{S}$, which annotates generators as $\boxed{a}^i$ with $i \in \{0,1\}$ and variables as $x^s$ with a stream $s$, is given inductively below. We lift $\beta$-reduction to stream-labeled terms by introducing a substitution case for stream-labeled variables: $x^s[M/x] = M^s$.

$$(\lambda x.\,N)^s = \lambda x.\,N^s \qquad\qquad (\boxed{a}.\,N)^{i\cdot s} = \boxed{a}^i.\,N^s$$

$$(N\,M)^s = N^s\,M \qquad\qquad (N \overset{a}{\oplus} M)^s = N^s \overset{a}{\oplus} M^s$$

**Definition 32.** *Projective reduction* $\rightarrow_\pi$ on stream-labeled terms is the rewrite relation given by

$$\boxed{a}^i.\,N \;\rightarrow_\pi\; \pi_i^a(N)\ .$$

Observe that in $N^s$ a generator that occurs under $n$ other generators on the spine of $N$, is labeled with the element of $s$ at position $n+1$. Generators in argument position remain unlabeled, until a $\beta$-step places them on the spine, in which case they become labeled by the new substitution case. We allow to annotate a term with a finite prefix of a stream, *e.g.* $N^i$ with a singleton $i$, so that only part of the spine is labeled. Subsequent labeling of a partly labeled term is then by $(N^r)^s = N^{r\cdot s}$ (abusing notation). To introduce streams via the external probabilistic sum, and to ignore an unused remaining stream after completing a probabilistic computation, we adopt the following equation.

$$N = N^0 + N^1$$

**Proposition 33.** *Projective reduction generalizes projective head reduction:*

$$H[\boxed{a}.\,N] \;=\; H[\boxed{a}^0.\,N] + H[\boxed{a}^1.\,N] \;\rightarrow_\pi\; H[\pi_0^a(N)] + H[\pi_1^a(N)]\;.$$

Returning to the interchangeability of probabilistic events, we refine (4) by exchanging the corresponding elements of the annotating streams:

$$\begin{array}{ccccc} (\boxed{a}.\,\boxed{b}.\,N)^{i\cdot j\cdot s} & = & \boxed{a}^i.\,\boxed{b}^j.\,N^s & \xrightarrow{\pi}\!\!\!\twoheadrightarrow & \pi_i^a(\pi_j^b(N^s)) \\ \sim & & & & = \\ (\boxed{b}.\,\boxed{a}.\,N)^{j\cdot i\cdot s} & = & \boxed{b}^j.\,\boxed{a}^i.\,N^s & \xrightarrow{\pi}\!\!\!\twoheadrightarrow & \pi_j^b(\pi_i^a(N^s)) \end{array} \tag{5}$$

Stream-labeling externalizes all probabilities, making reduction deterministic. This is expressed by the following proposition, that stream-labeling commutes with reduction: if a generator remains unlabeled in $M$ and becomes labeled after a reduction step $M \rightarrow N$, what label it receives is predetermined. The deep reason is that stream labeling assigns an outcome to each generator in a way that corresponds to a call-by-name strategy for probabilistic reduction.

**Proposition 34.** *If $M \rightarrow N$ by a step other than $\not\emptyset$ then $M^s \rightarrow N^s$.*

**Remark 35.** The statement is false for the $\not\emptyset$ rule $\boxed{a}.\,N \rightarrow_{\mathsf{p}} N$ $(a \notin \mathsf{fl}(N))$, as it removes a generator but not an element from the stream. Arguably, for this reason the rule should be excluded from the calculus. On the other hand, the rule is necessary to implement idempotence of $\oplus$, rather than just $\overset{a}{\oplus}$, as follows.

$$N \oplus N \;=\; \boxed{a}.\,N \overset{a}{\oplus} N \;\rightarrow_{\mathsf{p}}\; \boxed{a}.\,N \;\rightarrow_{\mathsf{p}}\; N \qquad \text{where } a \notin \mathsf{fl}(N)$$

The below proposition then expresses that projective reduction is an *invariant* for permutative reduction. If $N \rightarrow_{\mathsf{p}} M$ by a step (that is not $\not\emptyset$) on a labeled generator $\boxed{a}^i$ or a corresponding choice $\overset{a}{\oplus}$, then $N$ and $M$ reduce to a common term, $N \rightarrow_\pi P\; {}_\pi\!\!\leftarrow M$, by the projective steps evaluating $\boxed{a}^i$.

**Proposition 36.** *Projective reduction is an invariant for permutative reduction, as follows (with a case for $\mathsf{c}_2$ symmetric to $\mathsf{c}_1$, and where $D[\,]$ is a context).*

$$\begin{array}{ccc} \boxed{a}^i.\,C[N \overset{a}{\oplus} N] & \xrightarrow{\;\mathsf{p}\;} & \boxed{a}^i.\,C[N] \\ & \searrow_\pi \quad \mathsf{i} \quad {}_\pi\swarrow & \\ & \pi_i^a(C[N]) & \end{array} \qquad \begin{array}{ccc} \boxed{a}^i.\,C[(N_0 \overset{a}{\oplus} M) \overset{a}{\oplus} N_1] & \xrightarrow{\;\mathsf{p}\;} & \boxed{a}^i.\,C[N_0 \overset{a}{\oplus} N_1] \\ & \searrow_\pi \quad \mathsf{c}_1 \quad {}_\pi\swarrow & \\ & \pi_i^a(C[N_i]) & \end{array}$$

$$\begin{array}{ccc} \boxed{a}^i.\,C[D[N_0 \overset{a}{\oplus} N_1]] & \xrightarrow{\;\mathsf{p}\;} & \boxed{a}^i.\,C[D[N_0] \overset{a}{\oplus} D[N_1]] \\ & \searrow_\pi \quad \oplus\star \quad {}_\pi\swarrow & \\ & \pi_i^a(C[D[N_i]]) & \end{array}$$

$$\begin{array}{ccc} \lambda x.\,\boxed{a}^{\,i}.\,N & \xrightarrow{\ \mathsf{p}\ } & \boxed{a}^{\,i}.\,\lambda x.\,N \\ {\scriptstyle\pi}\downarrow & \square\lambda & \downarrow{\scriptstyle\pi} \\ \lambda x.\,\pi^a_i(N) & = & \pi^a_i(\lambda x.\,N) \end{array} \qquad \begin{array}{ccc} (\boxed{a}^{\,i}.\,N)M & \xrightarrow{\ \mathsf{p}\ } & \boxed{a}^{\,i}.\,NM \\ {\scriptstyle\pi}\downarrow & \square\mathsf{f} & \downarrow{\scriptstyle\pi} \\ \pi^a_i(N)\,M & = & \pi^a_i(N\,M) \end{array}$$

## 7 Call-by-value Interpretation

We consider the interpretation of a call-by-value probabilistic $\lambda$-calculus. For simplicity we will allow duplicating (or deleting) $\beta$-redexes, and only restrict duplicating probabilities; our *values* $V$ are then just deterministic—*i.e.* without choices—terms, possibly applications and not necessarily $\beta$-normal (so that our $\to_{\beta\mathsf{v}}$ is actually $\beta$-reduction on deterministic terms, unlike [9]). We evaluate the internal probabilistic choice $\oplus_{\mathsf{v}}$ to an external probabilistic choice $+$.

$$N ::= x \mid \lambda x.N \mid MN \mid M \oplus_{\mathsf{v}} N \qquad (\lambda x.N)V \to_{\beta\mathsf{v}} N[V/x]$$

$$V, W ::= x \mid \lambda x.V \mid VW \qquad M \oplus_{\mathsf{v}} N \to_{\mathsf{v}} M + N$$

The interpretation $[\![N]\!]_{\mathsf{v}}$ of a call-by-value term $N$ into $\Lambda_{\mathsf{PE}}$ is given as follows. First, we translate $N$ to a label-open term $[\![N]\!]_{\mathsf{open}} = \theta \vdash_L P$ by replacing each choice $\oplus_{\mathsf{v}}$ with one $\overset{a}{\oplus}$ with a unique label, where the label-context $\theta$ collects the labels used. Then $[\![N]\!]_{\mathsf{v}}$ is the *label closure* $[\![N]\!]_{\mathsf{v}} = \lfloor \theta \vdash_L P \rfloor$, which prefixes $P$ with a generator $\boxed{a}$ for every $a$ in $\theta$.

**Definition 37.** (Call-by-value interpretation) The *open interpretation* $[\![N]\!]_{\mathsf{open}}$ of a call-by-value term $N$ is as follows, where all labels are fresh, and inductively $[\![N_i]\!]_{\mathsf{open}} = \theta_i \vdash_L P_i$ for $i \in \{1, 2\}$.

$$\begin{array}{rcl} [\![x]\!]_{\mathsf{open}} & = & \vdash_L x \\ [\![\lambda x.N_1]\!]_{\mathsf{open}} & = & \theta_1 \vdash_L \lambda x.P_1 \end{array} \qquad \begin{array}{rcl} [\![N_1 N_2]\!]_{\mathsf{open}} & = & \theta_2 \cdot \theta_1 \vdash_L P_1 P_2 \\ [\![N_1 \oplus_{\mathsf{v}} N_2]\!]_{\mathsf{open}} & = & \theta_2 \cdot \theta_1 \cdot a \vdash_L P_1 \overset{a}{\oplus} P_2 \end{array}$$

The *label closure* $\lfloor \theta \vdash_L P \rfloor$ is given inductively as follows.

$$\lfloor \vdash_L P \rfloor = P \qquad \lfloor a \cdot \theta \vdash_L P \rfloor = \lfloor \theta \vdash_L \boxed{a}.\,P \rfloor$$

The *call-by-value interpretation* of $N$ is $[\![N]\!]_{\mathsf{v}} = \lfloor [\![N]\!]_{\mathsf{open}} \rfloor$.

Our call-by-value reduction may choose an arbitrary order in which to evaluate the choices $\oplus_{\mathsf{v}}$ in a term $N$, but the order of generators in the interpretation $[\![N]\!]_{\mathsf{v}}$ is necessarily fixed. Then to simulate a call-by-value reduction, we cannot choose a fixed context stream a priori; all we can say is that for every reduction, there is some stream that allows us to simulate it. Specifically, a reduction step $C[N_0 \oplus_{\mathsf{v}} N_1] \to_{\mathsf{v}} C[N_j]$ where $C[\,]$ is a call-by-value term context is simulated by the following projective step.

$$\ldots \boxed{a}^{\,i}.\,\boxed{b}^{\,j}.\,\boxed{c}^{\,k} \ldots D[P_0 \overset{b}{\oplus} P_1] \to_{\pi} \ \ldots \boxed{a}^{\,i}.\,\boxed{c}^{\,k} \ldots D[P_j]$$

Here, $[\![C[N_0 \oplus_{\mathsf{v}} N_1]]\!]_{\mathsf{open}} = \theta \vdash_L D[P_0 \overset{b}{\oplus} P_1]$ with $D[\,]$ a $\Lambda_{\mathsf{PE}}$-context, and $\theta$ giving rise to the sequence of generators $\ldots \boxed{a}.\boxed{b}.\boxed{c} \ldots$ in the call-by-value translation. To simulate the reduction step, if $b$ occupies the $n$-th position in $\theta$, then the $n$-th position in the context stream $s$ must be the element $j$. Since $\beta$-reduction survives the translation and labeling process intact, we may simulate call-by-value probabilistic reduction by projective and $\beta$-reduction.

**Theorem 38.** *If $N \twoheadrightarrow_{\mathsf{v},\beta\mathsf{v}} V$ then $[\![N]\!]^s_{\mathsf{v}} \twoheadrightarrow_{\pi,\beta} [\![V]\!]_{\mathsf{v}}$ for some stream $s \in \mathbb{S}$.*

## 8 Conclusions and Future Work

We believe our decomposition of probabilistic choice in $\lambda$-calculus to be an elegant and compelling way of restoring confluence, one of the core properties of the $\lambda$-calculus. Our probabilistic event $\lambda$-calculus captures traditional call-by-name and call-by-value probabilistic reduction, and offers finer control beyond those strategies. Permutative reduction implements a natural and fine-grained equivalence on probabilistic terms as internal rewriting, while projective reduction provides a complementary and more traditional external perspective.

There are a few immediate areas for future work. Firstly, within probabilistic $\lambda$-calculus, it is worth exploring if our decomposition opens up new avenues in semantics. Secondly, our approach might apply to probabilistic reasoning more widely, outside the $\lambda$-calculus. Most importantly, we will explore if our approach can be extended to other computational effects. Our use of streams interprets probabilistic choice as a *read* operation from an external source, which means other read operations can be treated similarly. A complementary treatment of *write* operations would allow us to express a considerable range of effects, including input/output and state.

### Acknowledgments

This work was supported by EPSRC Project EP/R029121/1 *Typed Lambda-Calculi with Sharing and Unsharing*. The first author is partially supported by the ANR project 19CE480014 PPS, the ERC Consolidator Grant 818616 DIAPASoN, and the MIUR PRIN 201784YSZ5 ASPRA. We thank the referees for their diligence and their helpful comments. We are grateful to Chris Barrett and—indirectly—Anupam Das for pointing us to Zantema and Van de Pol's work [27].
